# Identification and Antioxidant Abilities of Enzymatic-Transesterification (−)-Epigallocatechin-3-*O*-gallate Stearyl Derivatives in Non-Aqueous Systems

**DOI:** 10.3390/antiox10081282

**Published:** 2021-08-13

**Authors:** Chengyu Jiang, Li Wang, Xin Huang, Song Zhu, Chaoyang Ma, Hongxin Wang

**Affiliations:** 1 State Key Laboratory of Food Science and Technology, Jiangnan University, Wuxi 214122, China; jcy87injiangnan@163.com (C.J.); 6180112151@stu.jiangnan.edu.cn (L.W.); huangxin1728@163.com (X.H.); zhusong@jiangnan.edu.cn (S.Z.); machaoyang24@jiangnan.edu.cn (C.M.); 2 School of Food Science and Technology, Jiangnan University, Wuxi 214122, China

**Keywords:** EGCG stearyl derivatives, enzymatic transesterification, non-aqueous system, identification, antioxidant ability

## Abstract

Vinyl stearate was added to enzymatic transesterification of (−)-Epigallocatechin-3-*O*-gallate (EGCG) to enhance its lipophilicity and antioxidant ability in a non-aqueous system. The lipase DF “Amano” 15 was used as the catalyst. The optimal reaction conditions were: acetonitrile as the solvent, the molar ratio of vinyl stearate: EGCG as 3:1, an enzyme amount of 4.0% (ratio of substrate mass), and a reaction temperature and time of 50 °C and 96 h, respectively, achieving 65.2% EGCG conversion. HPLC–MS and NMR were used to determine the structure of EGCG stearyl derivative (3″,5″-2-O-stearyl-EGCG). The lipophilicity of EGCG stearyl derivatives (3.49 ± 0.34) was higher (5.06 times) than that of the parent EGCG (0.69 ± 0.08). Furthermore, EGCG stearyl derivatives had excellent lipid oxidation compared with BHT, BHA, and parent EGCG. The POVs of soybean oil with EGCG stearyl derivatives (18.17 ± 0.92 mEq/kg) were significantly reduced (by 62.5%) at 21 d compared with those of EGCG (48.50 ± 1.23 mEq/kg). These results indicate that EGCG derivatives have broad antioxidant application prospects in lipophilic environments/high-fat food.

## 1. Introduction

Lipid oxidation is the primary cause of flavor and nutrient loss in fat-containing foods, leading to adverse physiological reactions or food poisoning [1]. In recent years, some studies have shown that synthetic fat-soluble antioxidants, such as butylated hydroxytoluene (BHT), butylated hydroxyanisole (BHA), and tert-butylhydroquinone (TBHQ) have potential toxicity and carcinogenic effects [2,3,4]. Many consumers prefer natural ingredients to synthetic ones. Epigallocatechin-3-*O*-gallate (EGCG) is the main derivative of catechins with excellent biological activity [5]. However, EGCG has a polyhydroxy structure and hydrophilicity, limiting its application under hydrophobic conditions [6]. Nonetheless, the development of natural oil-soluble antioxidants, and the modification of the physical properties of known natural water-soluble antioxidants, have attracted a lot of attention.

Research on polyphenol modification (physical, chemical, and enzymatic) has advanced significantly in recent years. However, the particles and nanoparticles of polyphenols can easily undergo precipitation, demulsification, and delamination due to stirring, heating, and long-term storage [7]. Polyphenol derivatives obtained via chemical modification have poor selectivity and antioxidant activity [8]. The entire reaction process is affected by many factors, and generates unnecessary by-products and toxic waste [9]. Tobiason first detected enzymatic modification while analyzing molecular orbitals of catechin 3-*O*-acylated derivatives [10]. The process mainly occurs at the 3rd hydroxyl group of non-ester catechins and the B- and D-ring hydroxyl groups of ester catechins.

To date, various acyl donors have been associated with the acylation of flavonoids, where fatty acids are obtained due to their highest conversion degrees and reaction rates [11,12]. Enzymatic modification of EGCG mainly occurs at the 3,5-position of the hydroxyl group of the D- and B-ring of ester EGCG. The lipophilic derivatives of EGCG have an excellent partition coefficient (log *P* value). The log *P* value and the fat solubility gradually increase with the increasing number of hydroxyl substitutions [13]. However, the conversion yields of lipophilic derivatives of flavonoids decrease (from 62% to 27%) with the increasing fatty acid chain length [14]. Furthermore, long-chain EGCG derivatives are more stable, have stronger hydrophobicity, and have higher bioavailability than short-chain EGCG derivatives [15]. Moreover, higher lipophilicity can enhance the penetration of modified EGCG into the lipid bilayer of the cell membrane, promoting its absorption and utilization, and thereby increasing the expression of antioxidant activity in vivo [16]. In ARPE-19 cells, the monoalkylated EGCG (C18-EGCG) enhanced the protective effect against oxidative stress via the increase in lipid moiety [17]. Compared with short-chain fatty acids, the use of long-chain fatty acids can greatly improve lipid solubility and reduce the loss of phenolic hydroxyl groups, thus maintaining antioxidant activity.

Only a few studies have reported on the enzymatic molecular modification of EGCG via long-chain fatty acids. Herein, the lipophilic esterification of EGCG catalyzed by a new catalytic lipase enzyme was conducted in a non-aqueous solvent with vinyl stearate as an acyl donor. The effects of the enzyme source yield, substrate concentration, solvent system, enzyme addition, operating temperature, and reaction time on the conversion of EGCG stearyl derivatives were evaluated. HPLC–MS and NMR were used to assess the structural characteristics of EGCG stearyl derivatives. The lipophilicity and the antioxidant properties of the EGCG stearyl derivatives were determined and also compared with those of synthetic BHT, BHA, and TBHQ.

## 2. Materials and Methods

### 2.1. Materials

The immobilized enzyme of Novozym 435 (10,000 PLU/g), RMIM (275 IUN/g), TLIM (250 IUN/g), PSIM (500 U/g), and RM (275 IUN/g) were obtained from Novo Nordisk, Denmark. The immobilized enzyme of AY “Amano” 30SD and DF “Amano” 15 were acquired from Amano Enzyme (Jiangsu) Co., Ltd., (Shanghai, China). EGCG (purity 98%) was obtained from Gosun Biotechnologies Co., Ltd. (Hangzhou, Zhejiang, China). Vinyl stearate (purity 99%), methanol, acetone and other analytical reagents were obtained from Sinopharm Group Chemical Reagent Co., Ltd. (Shanghai, China).

### 2.2. Preparation of EGCG Derivatives via Different Reaction Parameters

The esterification reaction was conducted following the method described by Kontogianni et al. [18]. A certain amount of vinyl stearate (different molar ratios) was mixed with EGCG (100 mM) in 20 mL of organic solvents. The reaction was initiated by seven immobilized lipases, stirred and incubated at 30–55 °C for 12–96 h until the end of the reaction. The enzyme was then filtrated out. EGCG stearyl derivatives were stored in a 4 °C refrigerator for further experiments.

### 2.3. Determination of Conversion of EGCG Derivatives

A high-performance liquid chromatography (HPLC) method with a Waters 2998 Diode Array Detector (DAD), an Agilent SB-C18 column (150 × 4.6 mm, 5 μm), and a Waters 600 pump was used to assess the conversion of EGCG derivatives. The mobile phase contained 0.1% trifluoroacetic acid (A) and acetonitrile (B) with a gradient curve of 5–100% (B) for 0–20 min, 100% (B) for 20–25 min, 100–5% (B) for 25–30 min, and 5% (B) for 30–35 min at a flow rate of 1 mL/min. The injection volume was 5 μL at 280nm. The conversion of EGCG was calculated as follows:

Tr (%) = (A_1_ − A_0_)/A_1_ × 100%

where Tr indicates the conversion of EGCG (%); A_0_ and A_1_ represent the content of EGCG in the sample and the standard (µg/mL), respectively.

### 2.4. Purification and Identification of EGCG Stearyl Derivatives

The purification was performed via the semipreparative HPLC system with a Waters 1525 pump, a H&E SP ODS-A column, and a Waters 2998 DAD. The mobile phase contained 0.1% acetic acid (A) and methanol (B) with a linear gradient of 5−100% B for 0–30 min at a flow rate of 5 mL/min and wavelength of 280 nm. The injection volume was 1 mL.

The MALDI SYNAPT MS system (Waters, San Jose, MA, USA) was used as follows: positive mode, 400 °C of desolvation temperature, 3.5 kV of capillary voltage, 30 psi of nebulizer, and 5 min of dwell time. A nuclear magnetic resonance (NMR) spectrometer (Bruker BioSpin GmbH, Rheinstetten, Germany) was used to assess the grafting position of hydroxy for chemical shifts analysis of EGCG derivatives to standard EGCG.

### 2.5. Determination of 1-Octanol/Water Partition Coefficient

Lipophilicity was measured as described by Chen and Yu [19]. Briefly, the deionized water and octanol were thoroughly stirred for 24 h. The sample (0.2 µmol) was dissolved in 5 mL of upper phase (presaturated octanol). Deionized water (5 mL) was then added, vortexed and allowed to stand for separation. The absorbance of the upper phase (C_0_) and the bottom phase (C_w_) was measured at 280 nm. Log *P* was calculated using the following equation:

Log P = log (C_0_/C_w_).

where C_0_ and C_w_ indicate the sample in the upper bottom phases, respectively.

### 2.6. Determination of Radical Scavenging Ability of 2,2-Diphenylpicrylhydrazyl (DPPH)

The radical scavenging activity of DPPH was determined using the method described by Gordon et al. [20], with some modifications. In brief, a mixture of 0.1 mL of the sample (1 mg/mL) and 3.9 mL of DPPH (25 mg/L) was placed in the dark for 30 min and measured at 517 nm (UV-1800PC, MAPADA, Shanghai, China). A control was also analyzed. The radical scavenging ability was calculated as follows:

DPPH radical scavenging activity (%) = [(A_C_ − A_S_)/A_C_] × 100%

where A_S_ indicates the value with the sample, while A_C_ shows the value without a sample.

### 2.7. Determination of Radical Scavenging Ability of 2,2-Azino-bis (3-Ethylbenzothiazoline-6-sulfonate) (ABTS)

The radical scavenging activity of ABTS was measured using the method described by Asikin et al. [21]. K_2_S_2_O_8_ (88 μL) (140 mM) and 5 mL of ABTS solution (7 mM) were mixed and put in the dark for 16 h. The samples (1 mg/mL) were diluted with ethanol to 1180 μL, then 20 μL of ABTS solution was added. The absorbance was measured after 3 min at 734 nm. A blank was also analyzed. The radical scavenging ability was calculated as follows:

ABTS radical scavenging ability (%) = [(A_C_ − A_S_)/A_C_] × 100%

where A_S_ indicates the value with the sample, and A_C_ shows the value without a sample.

### 2.8. Reducing Power Assay

The reducing power assay was conducted as described by Gülçin et al. [22]. In brief, a mixture of the sample (250 μL), KH_2_PO_4_ buffer (250 μL, pH7.5), and potassium ferricyanide (1% (*w*/*v*), 250 μL) was bathed at 50 °C for 20 min. Trichloroacetic acid (10% (*w*/*v*), 250 μL) was then added and centrifuged, and the supernatant was obtained. A mixture of the supernatant (500 μL), distilled water (500 μL), and ferric chloride (0.1% (*w*/*v*), 100 μL) was placed in darkness for 15 min. Absorbance was measured at 700 nm.

### 2.9. Determination of Hydroxyl Antioxidant Ability

The antioxidant ability of hydroxyl was determined using the method described by Kong et al. [23]. In brief, a mixture of the sample (1 mg/mL), 1.0 mL FeSO_4_ (6.0 mM), and 1.0 mL H_2_O_2_ (2.5 μM) was incubated at 37 °C for 10 min. Salicylic acid (6.0 mM) (1.0 mL) was then added to initiate the reaction and bathed at 37 °C for 30 min. The absorbance was determined at 500 nm. The antioxidant ability of hydroxyl was calculated as follows:

Hydroxyl antioxidant ability (%) = [(A_0_ − (A_1_ − A_2_)/A_0_] × 100%

where A_0_, A_1_, and A_2_ indicate the absorbance of the control, sample, and reagent without sodium salicylate, respectively.

### 2.10. Determination of Oxygen Radical Absorbance Capacity (ORAC)

The ORAC assay was conducted as described by Re et al. [24]. The sample (20 μL) was mixed with fluorescein (160 μL, 1.6 nM). AAPH (306 μM, 20 μL) was then added to initiate the reaction. A fluorescence plate reader (Thermo Fisher Scientific, Waltham, MA, USA) was used to measure the fluorescence every 2 min for 2 h at 538 nm of emission wavelength and 485 nm of excitation wavelength.

### 2.11. Determination of Lipid Oxidation at Storage

The sample (6 mg) was mixed with 30 g of soybean oil, vortexed, and kept at 60 °C for 21 d. The peroxide values (POV) were measured every 3 d according to the GB/T5009.227-2016. In brief, a mixture of test material (2 g), chloroform-glacial acetic acid (30 mL), and saturated potassium iodide (1 mL) were placed in darkness for 3 min. Water (50 mL) was added, then the mixture was titrated with sodium thiosulfate (10 mM) immediately until a light yellow color appeared. A starch indicator (1 mL) was added, and the mixture titrated until the blue color disappeared. A blank was prepared under the same conditions. The POV was calculated as follows:

$$X=\frac{(V-V0)*c*0.1269}{m}*100\%$$

where X indicates the mequiv of active oxygen per kilogram of soybean oil; V and V_0_ show the volume of sodium thiosulfate in the sample and the blank, respectively; c indicates the concentration of sodium thiosulfate; m shows the quality of the test material; 0.1269 is the quality of iodine equivalent to 1 mL of sodium thiosulfate solution; and 100 is the conversion factor.

### 2.12. Statistical Analysis

Each experiment had three replicates. The results were expressed as mean ± standard deviation (SD). A one-way analysis of variance (ANOVA) was used to determine the significant differences between the multiple groups, with LSD and HSD methods in SPSS22.0. *p* < 0.05 was considered a significant difference.

## 3. Results and Discussion

### 3.1. Effect of Various Extraction Factors on the Synthesis of EGCG Derivatives

**Figure 1:**
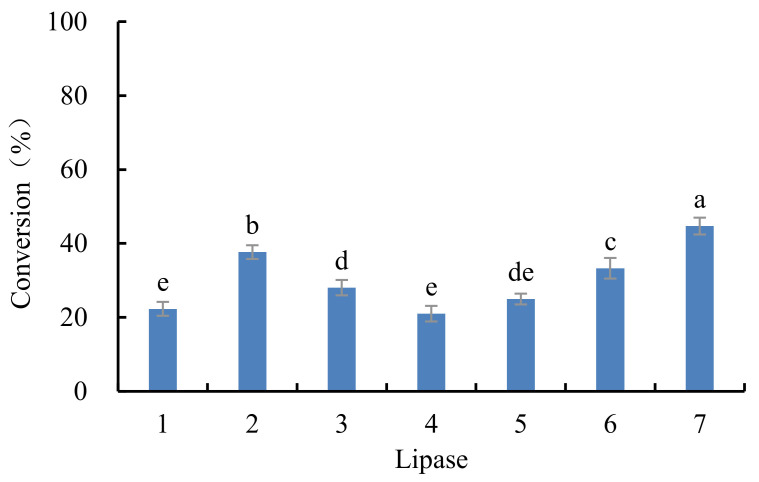
The conversion of EGCG by different lipases (reaction conditions: EGCG, 1 mmol; vinyl stearate, 3 mmol; enzyme, 5.0% (*w*/*w* of all substrates)); reaction temperature, 40 °C; reaction time, 48 h in acetonitrile. 1: Novozym 435; 2: TLIM; 3: PSIM; 4: RMIM; 5: RM; 6: AY “Amano” 30SD; 7: DF “Amano” 15. Bars with different letters are significantly different at *p* < 0.05.

Enzymatic modification has mild reaction conditions and specific reaction characteristics [25]. Conversions of EGCG catalyzed by seven different lipases are shown in Figure 1. Lipase DF “Amano” 15 had the highest catalytic ability of 44.7%, indicating high activity and stability, similar to lipase-catalyzed esterification [26]. Lipase *Rhizomucor miehei* (RMIM) and Novozym 435 (*Candida antarctica*) are selective to short and medium fatty acids [27], while DF”Amano” 15 (*Rhizopus oryzae*) may present high activity on a long alkyl chain.

**Figure 2:**
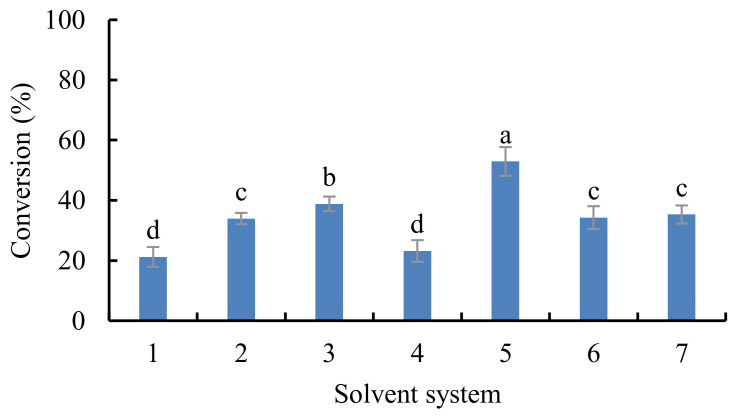
Conversion of EGCG by different solvent systems (reaction conditions: EGCG, 1 mmol; vinyl stearate, 3 mmol; enzyme, 5.0% lipase DF “Amano” 15 (*w*/*w* of both substrates)); reaction temperature, 50 °C; reaction time, 48 h in different solvent systems. 1: methanol; 2: ethanol; 3: isopropanol; 4: isopentanol; 5: acetonitrile; 6: acetone; 7: ethyl acetate. Bars with different letters are significantly different at *p* < 0.05.

Biocatalysts are more stable in non-polar solvents than polar solvents [28]. Hazarika, et al. [29] showed that solvents with small log *P* values could enhance the conversion. The conversion of EGCG (catalyzed by DF “Amano” 15) in various organic solvents is shown in Figure 2. Lipase-catalyzed esterification of EGCG in acetonitrile had the highest conversion (52.9%).

**Figure 3:**
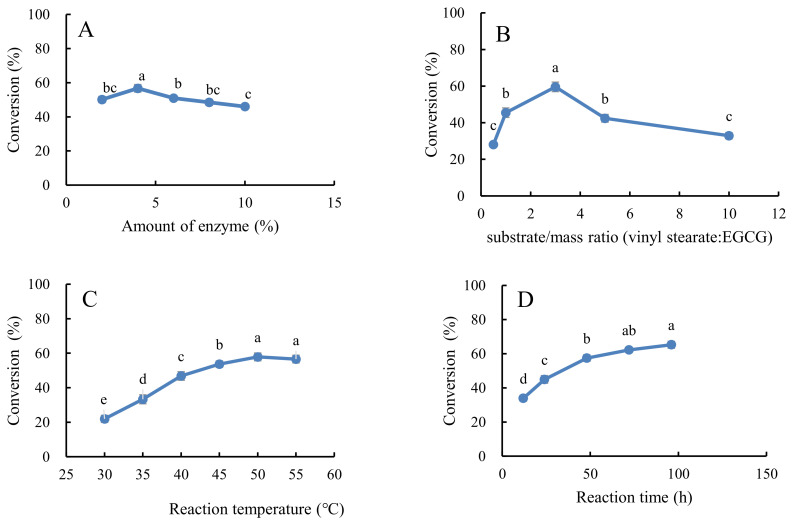
The effects of enzyme usage level with enzyme amount (lipase DF “Amano” 15, % *w*/*w* of both substrates). (**A**), substrate mass ratio (**B**), reaction temperature (**C**), and reaction time (**D**) on the conversion of EGCG. Values followed by different letters within the same line are significantly different (*p* < 0.05).

The effect of enzyme amount (lipase DF “Amano” 15) on the synthesis of EGCG derivatives in acetonitrile is shown in Figure 3A. The conversion increased with the increase in enzyme amount (from 2.0% to 4.0%), but decreased with further increases in the enzyme amount (from 4.0% to 10.0%) (*p* < 0.05). Yang et al. [30] have suggested that the enzyme particles tend to aggregate at high enzyme concentrations, which is not conducive for effective enzyme and substrate collision. These results suggest that 4.0% was the optimal concentration of lipase.

The increased ratio of vinyl stearate to EGCG from 1:1 to 3:1 increased the conversion of EGCG from 28.1 ± 1.1% to 59.6 ± 2.5%. The conversion then decreased to 32.9 ± 1.7% at the ratio of 10:1 (Figure 3B). These results indicate that excess vinyl stearate may inhibit the catalytic ability of the lipase DF “Amano” 15. Increased EGCG concentration reduced the substrate inhibition, thus enhancing ester synthesis.

The conversion showed a positive trend with reaction temperature at a certain reaction time (Figure 3C). The maximum conversion of EGCG occurred at (57.9 ± 2.3%) at 50 °C. However, it slightly decreased at 55 °C (56.6 ± 2.2%), possibly due to the esterification of EGCG, which can change hydrolysis direction under high temperatures [13]. High temperatures enhance the interaction between enzymes and substrates [31]. High temperatures can also lead to a loss of enzyme activity by destroying its active conformation. Therefore, a suitable temperature is crucial for the enzyme-catalyzed reaction.

Although the reaction time was positively related to the conversion of EGCG (Figure 3D), the increasing trend significantly decreased after 48 h. A slight increase in the conversion was observed from 48 to 96 h (from 57.4 ± 1.9% to 65.2 ± 2.0%). Herein, a vinyl stearate and EGCG ratio of 3:1 achieved a conversion of 65.2% after a 4.0% addition of lipase DF “Amano” 15 in acetonitrile system at 50 °C for 96 h.

### 3.2. Identification and Structure Analysis of EGCG Stearyl Derivatives

**Figure 4:**
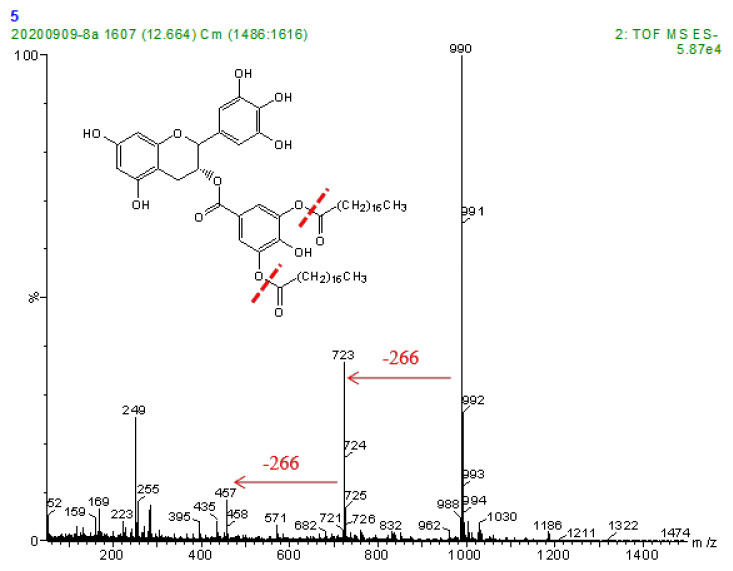
Mass spectra of EGCG stearyl derivatives.

A semi-preparative high-performance liquid chromatography system was used to obtain the solution of the EGCG stearyl derivatives. The purified EGCG stearate derivatives (purity: 78.5%) were obtained at 22.458 min and are shown in Figure S1. The MS spectra of EGCG derivatives are shown in Figure 4. The parent EGCG had a molecular weight of 457 Da, and that of the EGCG stearyl derivatives increased by 266 Da for each additional vinyl stearate group. The mono-stearyl and di-stearyl EGCG peaked at 723 Da and 990 Da, respectively.

The results of the NMR analysis for parent EGCG and di-stearyl EGCG are shown in Table 1. (1) Parent EGCG were as follows: 1H NMR (400 MHz, Methanol-*d*_4_) δ 6.97 (s, 2H), 6.52 (s, 2H), 5.98 (s, 2H), 5.55 (ddd, *J* = 4.5, 2.7, 1.4 Hz, 1H), 4.99 (s, 1H), 3.00 (dd, *J* = 17.3, 4.6 Hz, 1H), 2.86 (dd, *J* = 17.4, 2.7 Hz, 1H). 1H NMR (400 MHz, Methanol-*d*_4_) δ 6.97 (H-2″, 6″), 6.52 (H-2′, 6′), 5.98 (H-6, 8), 5.55 (H-3), 4.99 (H-2), 3.00 (dd, *J* = 17.3, 4.6 Hz, 1H), 2.86 (H-4). 13C NMR (101 MHz, Methanol-*d*_4_) δ 166.26 (COO), 156.47 (C-7, 9), 155.83 (C-5), 145.29 (C-3″, 5″), 144.89 (C-3′, 5′), 138.39 (C-4″), 132.40 (C-4′), 129.42 (C-1″), 120.14 (C-1′), 108.88 (C-2″, 6″), 105.50 (C-2′, 6′), 98.04 (C-10), 95.15 (C-8), 94.49 (C-6), 77.22 (C-2), 68.54 (C-3), 25.43 (C-4).

(2) Di-stearoyl EGCG were as follows: 1H NMR (400 MHz, Methanol-*d*_4_) δ 7.29 (m, 1H), 7.09 (h, *J* = 2.6 Hz, 0H), 6.97 (d, *J* = 4.7 Hz, 1H), 6.86 (d, *J* = 2.1 Hz, 0H), 6.68 (d, *J* = 2.1 Hz, 0H), 6.59 (d, *J* = 6.9 Hz, 1H), 6.51 (d, *J* = 4.5 Hz, 0H), 5.98 (dd, *J* = 3.4, 1.9 Hz, 0H), 5.57 (m, 1H), 4.99 (s, 0H), 3.01 (m, 1H), 2.88 (dt, *J* = 17.5, 2.7 Hz, 1H), 2.57 (m, 2H), 2.41 (t, *J* = 7.4 Hz, 1H), 2.31 (dt, *J* = 12.2, 7.4 Hz, 0H), 2.17 (s, 2H), 1.68 (m, 2H), 1.45 (q, *J* = 6.5, 5.7 Hz, 1H), 1.31 (d, *J* = 3.7 Hz, 49H), 0.92 (t, *J* = 6.7 Hz, 5H). 13C NMR (101 MHz, Methanol-*d*_4_) δ 172.44 (COO), 170.86 (COO), 166.21 (COO), 156.42 (C-7, 9), 155.55 (C-5), 149.75 (C-3″, 5″), 145.11 (C-3′, 5′), 136.74 (C-4″), 132.41 (C-4′), 129.28 (C-1″), 120.03 (C-1′), 108.89 (C-2″,6″), 105.51 (C-2′, 6′), 97.98 (C-10), 95.01 (C-8), 94.30 (C-6), 76.88 (C-2), 68.38 (C-3), 33.19 (-CH2), 31.70 (-CH2), 29.42 (-CH2), 29.39 (-CH2), 29.29 (-CH2), 29.10 (-CH2), 28.75 (-CH2), 25.36 (C-4), 24.57 (-CH2), 24.30 (-CH2), 22.83 (-CH2), 22.36 (-CH2), 13.08 (-CH3).

**Figure 5:**
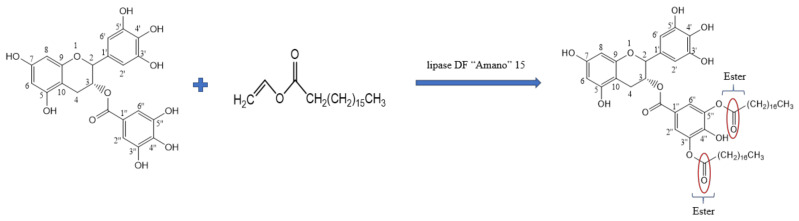
Schematic representation of enzymatic synthesis of di-stearyl EGCG.

The downfield shifts of the EGCG derivatives are shown in Table 1. The 1H NMR results showed that the D-ring of the EGCG molecule contained an acetyl group. The 13C NMR results showed the specific position of acylation. The remarkable upfield shift (Δδ 1.65) of C-4″, and the significant downfield shift (Δδ 4.46) of C-3″ and C-5″ may be the acylation sites (Table 1). The carbons in the A-ring and C-ring did not have downfield or upfield shifts. Therefore, the structure of the di-stearyl EGCG was identified as 3″,5″-2-O-stearoyl epigallocatechin gallate (Figure 5).

### 3.3. Lipophilicity of EGCG Stearyl Derivatives

Many hydrophilic bioactive compounds have been lipophilized for use in a lipophilic system [32,33,34]. The log *P* values of parent EGCG and EGCG stearyl derivatives are shown in Table 2 (0.69 ± 0.08 and 3.49 ± 0.34, respectively) (*p* < 0.05). The log *p* value of EGCG stearyl derivatives was higher than that of the parent EGCG, as the stearyl group replaced the hydroxyl group. The enhanced lipophilicity of EGCG derivatives enhances the application of EGCG and other water-soluble antioxidants in the oxidation inhibition of fatty foods. These results further indicate that lipid acylation of EGCG significantly improves the lipid solubility of EGCG. The cell membrane permeability also increases due to increased fat solubility, thus increasing antioxidant activity [35].

### 3.4. Antioxidative Activities of EGCG Stearyl Derivatives and Synthetic Antioxidants

**Figure 6:**
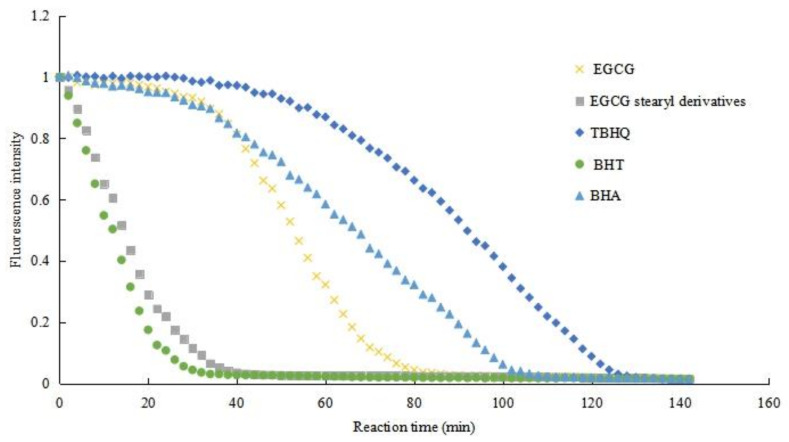
Oxygen radical absorbance capacity of EGCG, EGCG stearyl derivatives, and synthetic antioxidants.

Antioxidant assay methods can be divided into two categories based on chemical reaction mechanisms: electron transfer (ET) and hydrogen atom transfer (HAT) [36]. The antioxidative properties of EGCG, EGCG stearyl derivatives, BHT, BHA, and TBHQ are shown in Table 3. The DPPH has a deep violet color in the solution. Antioxidants can reduce the DPPH radicals to a yellow substance. The DPPH abilities of EGCG, EGCG stearyl derivatives, BHT, BHA, and TBHQ (1 mg/mL) were 90.89%, 51.49%, 9.74%, 72.38%, and 92.99%, respectively. ABTS can be converted to free radical cation by adding sodium persulfate and absorbed at 734 nm. Herein, the ABTS abilities of EGCG, EGCG stearyl derivatives, BHT, BHA, and TBHQ were 90.36%, 65.16%, 56.30%, 60.06%, and 63.01%, respectively. The reducing power assay is a typical ET-based method, where antioxidants in acidic media reduce the iron (Fe^3+^) ligand complex to a dark blue ferrous (Fe^2+^) complex, and have a mix absorption at 700 nm. The reducing power values of EGCG, EGCG stearyl derivatives, BHT, BHA, and TBHQ were 2.845, 1.857, 1.710, 2.260, and 2.689, respectively. Hydroxyl radicals can easily be generated via ultraviolet decomposition of H_2_O_2_ and have strong reactivity. Herein, the hydroxyl radical scavenging activities of EGCG, EGCG stearyl derivatives, BHT, BHA, and TBHQ were 30.71%, 16.84%, 11.33%, 7.18%, and 73.86%, respectively. The ORAC assay was used to determine the quenching effect of free radicals on fluorescent probes. Antioxidants can inhibit the fluorescence changes caused by free radicals. The results of AAPH-mediated FL oxidation of EGCG, EGCG derivatives, BHT, BHA, and TBHQ (1 mg/mL), are shown in Figure 6.

Hydrophobicity is the main factor affecting the activity of phenolic compounds [37,38]. Although the antioxidant ability of EGCG stearyl derivatives was higher than or equal to that of BHA and BHT, it was lower than that of TBHQ. These results are similar to that of modified epicatechin, which has strong antioxidant and free radical scavenging abilities [39]. The coupling of alkyl groups to phenolic compounds reduced the free radical scavenging ability, indicating that the increase in chain length and the enhanced hydrophobicity do not necessarily improve the antioxidant capacity in emulsions [40]. Furthermore, Laguerre et al. [41] have shown that the increased hydrophobicity of antioxidants can drive the active site into the lipid phase, thus decreasing antioxidant ability.

**Figure 7:**
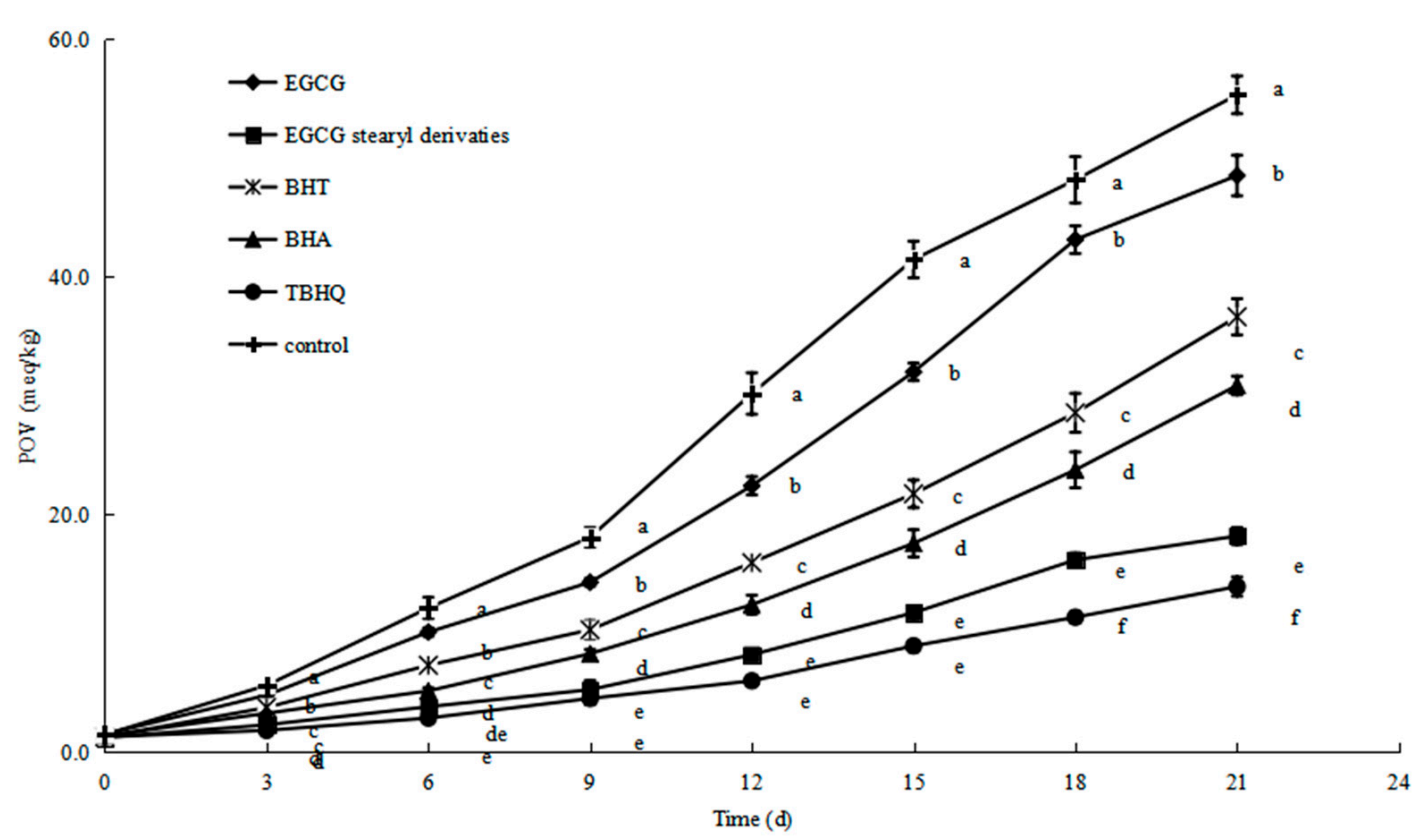
The POV of soybean oil containing EGCG, EGCG stearyl derivatives, and synthetic antioxidants under storage. Values followed by different letters within the same time are significantly different (*p* < 0.05).

POV is a widely used indicator of oil stability as a measure of hydrogen peroxide concentration during the oil oxidation stage. The POVs of soybean oil with the parent EGCG, stearyl EGCG, BHT, BHA, and TBHQ are shown in Figure 7. The POVs of soybean oil samples with EGCG, EGCG stearyl derivatives, BHT, BHA, and TBHQ were 48.50 ± 1.23, 18.17 ± 0.92, 36.61 ± 0.95, 30.84 ± 0.92, and 13.92 ± 0.70 meq/kg, respectively. The above POVs were significantly different from soybean oils (55.29 ± 1.59 meq/kg) at the 21 d (*p* < 0.05). Overall, oxidation reactions in bulk oil systems occur at the oil–air interface and/or the dissolved oxygen within the oil interface [42]. These results showed that the lipid oxidation inhibition ability was stronger in EGCG stearyl derivatives than in EGCG, but slightly less strong than in TBHQ. This finding suggests that EGCG stearyl derivatives may promote oil stability by activating the free radicals and phenolic hydroxyl reaction, whereas TBHQ uses phenyl ring [43]. The development of water-soluble natural polyphenols as EGCG stearyl derivatives could produce safe food additives with antioxidant properties, as an alternative to synthetic antioxidants which have been shown to have potential hazards.

## 4. Conclusions

We found that lipase DF “Amano” 15 had the highest activity and efficiency in the transesterification reaction between EGCG and vinyl stearate. A vinyl stearate: EGCG ratio of 3:1 achieved a 65.2% conversion of EGCG after 96 h at 50 °C in an acetonitrile system. HPLC–MS, and NMR identified the structure of the EGCG derivatives as 3″,5″-2-O-stearyl-EGCG. The EGCG stearyl derivatives have excellent lipophilicity. Moreover, their antioxidant ability and lipid oxidation inhibition abilities are higher than or equal to BHT, BHA, and parent EGCG. Compared with the chemical esterification of EGCG, which often produces toxic waste and unnecessary by-products, the enzymatic modification of EGCG is characterized by mild reaction conditions, environmental protection, and economic benefits. Finally, the excellent lipophilicity and antioxidative activity in the lipophilic system indicate that these derivatives have broad antioxidant application prospects in lipophile environments/high-fat food, and are suitable for use in the food, medicine and cosmetic industries.

## Figures and Tables

**Table 1:** ^1^H and ^13^C Chemical Shifts (δ) of EGCG and Di-stearyl EGCG.

	EGCG		Di-Stearyl EGCG	
C/H Position	^1^H	^13^C	^1^H	^13^C
2	4.99	77.22	4.99	76.88
3	5.55	68.54	5.57	68.38
4	3.00	25.43	3.01	25.36
	2.86		2.88	
5		155.83		155.55
6	5.98	94.49	5.98	94.30
7		156.47		156.42
8	5.98	95.15	5.98	95.01
9		156.47		156.42
10		98.04		97.98
1’		120.14		120.03
2’	6.52	105.50	6.51	105.51
3’		144.89		145.11
4’		132.40		132.41
5’		144.89		145.11
6’	6.52	105.50	6.51	105.51
1”		129.42		129.28
2”	6.97	108.88	6.86	108.89
3”		145.29		149.75
4”		138.39		136.74
5”		145.29		149.75
6”	6.97	108.88	6.86	108.89
COO		166.26		166.21
				170.68
				172.44

Proton and carbon in alkyl chain of incorporated fatty acids are not listed.

**Table 2:** Lipophilicity of EGCG Stearyl Derivatives and Parent EGCG.

	Octanol/Water Partition Coefficient (logP)		
EGCG Stearyl Derivatives	3.49	±	0.34 ^a^
EGCG	0.69	±	0.08 ^b^

Values of three replicates (mean ± SD) with different letters were significantly different at *p* < 0.05.

**Table 3:** Antioxidant Abilities of EGCG, EGCG Stearyl Derivatives, and Synthetic Antioxidants.

	DPPH Inhibition(%)	ABTS Inhibition (%)	Reducing Power (OD)	Hydroxyl Inhibition (%)
EGCG	90.89 ± 2.56 ^a^	90.36 ± 3.30 ^a^	2.845 ± 0.086 ^a^	30.71 ± 1.90 ^b^
EGCG Stearyl Derivatives	51.49 ± 1.80 ^c^	65.16 ± 1.28 ^b^	1.857 ± 0.044 ^c^	16.84 ± 1.18 ^c^
BHT	9.74 ± 0.65 ^d^	56.30 ± 2.30 ^c^	1.710 ± 0.033 ^c^	11.33 ± 0.80 ^d^
BHA	72.38 ± 2.31 ^b^	60.06 ± 2.88 ^bc^	2.260 ± 0.063 ^b^	7.18 ± 0.87 ^e^
TBHQ	92.99 ± 2.06 ^a^	63.01 ± 1.64 ^b^	2.689 ± 0.061 ^a^	73.86 ± 2.87 ^a^

Values of three replicates (mean ± SD) with different letters were significantly different at *p* < 0.05.

## Data Availability

Data is contained within the article and Supplementary Materials.

## Supplementary Materials

The following are available online at https://www.mdpi.com/article/10.3390/antiox10081282/s1. Figure S1. High performance liquid chromatography of the purification of EGCG stearyl derivatives.
