# Supplementary material for: Identification and Antioxidant Abilities of Enzymatic-Transesterification (−)-Epigallocatechin-3-O-gallate Stearyl Derivatives in Non-Aqueous Systems
Source: Antioxidants (Basel). 2021 Aug 13;10(8):1282. doi: 10.3390/antiox10081282 (PMC8389292; doi:10.3390/antiox10081282)
Supplement: Supplementary file 1 [file antioxidants-10-01282-s001.zip › antioxidants-1287228-supplementary.pdf]

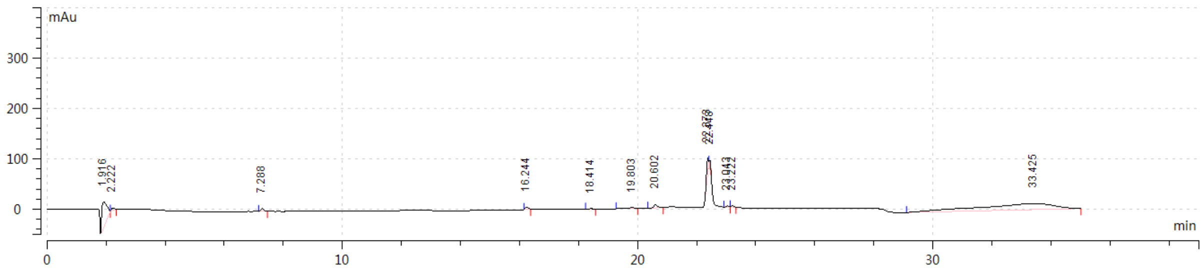

Supplementary figure s1. High performance liquid chromatography of the purification of EGCG stearyl derivatives.
